# Grady Hospital Affair Finally Settled

**Published:** 1908-03

**Authors:** 


### GRADY HOSPITAL AFFAIR FINALLY SETTLED.

---

It is with genuine pleasure we note the settlement of the disturbance over Grady Hospital Wards, and it is to be hoped that the energy formerly expended in the wrangling will now be employed in a firm and persistent effort to maintain peace and manage the hospital in a manner just and above reproach.

The present arrangement appears fair to an unprejudiced observer, and this view obtains still further support from the fact that neither side is fully satisfied, for each wanted a little more.

The wards are now open to students and the staff is to be increased from thirteen to eighteen; six are to be selected from the College of Physicians and Surgeons, six from the Atlanta School of Medicine, and six from the physicians not affiliated with any college. This is manifestly fair, and will enable the students who attend the Medical Colleges of Atlanta to obtain the benefit the abundant clinical material of Grady Hospital will afford for scientific observation and study. The additional care required to clear up obscure or difficult cases will result in direct good to the patients, for they cannot be demonstrated properly until a correct diagnosis has been made, and upon this hinges the success in treatment, be it medical or surgical.

Let all pull together for the good of Grady Hospital, her patients and the medical colleges, embued with scientific enthusiasm for investigation and advancement, and there need be no more serious dissention.

### THE PROHIBITION LAW.

Elsewhere in this issue will be seen a letter from Dr. Cartledge, taking exceptions to our editorial in the February number, in which we regretted that the recent prohibition law had been so hastily prepared that it did not exclude legitimate pharmaceutical preparations, that according to literal wording of the bill, cannot now be sold without an infringement of the law. Dr. Cartledge lays stress upon the claim that the drugs containing alcohol in any quantity also contain active ingredients which would poison an individual before the state of intoxication could be reached.

That this view is erroneous can be proven by reference to a few well known preparations, which, according to the wrapper on the bottles, contain alcohol as follows: Liquid Peptonoids, 12 per cent.; Hemaboloids, 17 per cent.; Stearns' Wine of Cod Liver Oil, 20 per cent.; Waterbury's Cod Liver Oil, 10 per cent., and a number of other similar preparations, now in constant use, which contain no ingredients more active than alcohol. Many of the so-called "bitters" also contain large quantities of alcohol, and other principles in quantities so small that the intoxicating effect may be obtained as it could be with liquid peptonoids, etc., without incurring any other deleterious effects.

We do not claim that any such drugs are now extensively used for such purposes, but according to the law, after one person has become drunk on them, no matter how limited his capacity for imbibing intoxicants, a strict enforcement of the law would exclude their sale.

